# Germacrone Regulates HBXIP-Mediated Cell Cycle, Apoptosis and Promotes the Formation of Autophagosomes to Inhibit the Proliferation of Gastric Cancer Cells

**DOI:** 10.3389/fonc.2020.537322

**Published:** 2020-11-10

**Authors:** Xing Fang, TingFei Tan, BeiBei Gao, YingLi Zhao, TingTing Liu, Quan Xia

**Affiliations:** ^1^ Department of Pharmacy, The First Affiliated Hospital of Anhui Medical University, Hefei, China; ^2^ Department of Pharmacy, The Second People’s Hospital of Hefei, Hefei, China; ^3^ Department of Pharmacy, The Grade 3 Pharmaceutical Chemistry Laboratory of State Administration of Traditional Chinese Medicine, Hefei, China

**Keywords:** cell cycle, autophagy, apoptosis, hepatitis B X-interacting protein, germacrone

## Abstract

Germacrone, a monocyclic sesquiterpene, exerts marked antitumor effects in a variety of cancers, including hepatocellular carcinoma, gastric cancer, and breast cancer. However, the mechanism underlying the effects of germacrone on gastric cancer remains unclear. In this study, we show that germacrone inhibited gastric cancer cell proliferation in a dose-dependent manner, and induced G0/G1-phase cell cycle arrest and apoptosis in these cells. Moreover, germacrone increased the expression of LC3II/LC3I. And LC3II/LC3I was significant increased after germacrone treatment compared with germacrone and bafilomycin A1 (Baf A1) treatment, which suggested germacrone promoted the formation of autophagosomes. Proteomic analysis was then used to identify molecular targets of germacrone in gastric cancer. A total of 596 proteins were screened, and the top hit was identified as late endosomal/lysosomal adaptor and MAPK and MTOR activator 5 (LAMTOR5, also named HBXIP). Overexpression of HBXIP delayed the germacrone-induced cell cycle arrest, induction of apoptosis, and inhibition of autophagy. Combined, our results indicate that germacrone suppresses gastric cancer cell proliferation by inhibiting HBXIP, and this process is related to G0/G1-phase arrest and apoptosis.

## Introduction

Gastric cancer is a leading cause of cancer-related deaths worldwide, and the prognosis for these patients remained dismal (1–3). The current clinical treatments for gastric cancer include surgery and chemotherapy; however, resistance of gastric cancer cells to conventional drugs has gradually increased, resulting in gastric cancer development and recurrence after surgery (4–6). Consequently, it is imperative that new and effective preventive drugs to treat human gastric cancer are identified.

**Figure 1:**
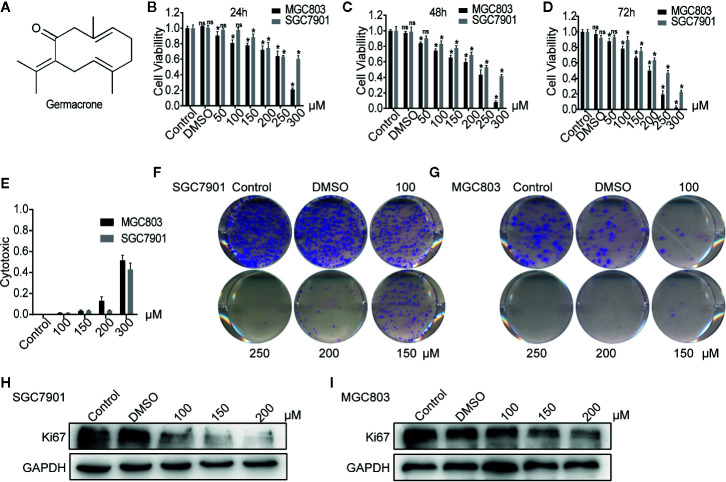
Germacrone inhibited the proliferation of gastric cancer cells in a dose-dependent manner. **(A)** The structural formula of germacrone. **(B–D)** The MTT assay assessing changes in cell viability after 24, 48, and 72 h of germacrone treatment (0, 50, 100, 150, 200, 250, and 300 µM). **(E)** Detection of lactate dehydrogenase (LDH) 24 h after germacrone treatment to evaluate its cytotoxicity. **(F, G)** Colony formation assay after 24 h of germacrone treatment (100, 150, and 200 µM). **(H, I)** Western blot analysis of KI67 protein expression. Data are the means ± SD of 3 experiments. *P < 0.05; ns, not significant.

*Curcuma zedoaria* is an important traditional herb that is widely used as a herbal medicine in China, India, and other Asian countries. It is indicated to exert antitumor effects in breast cancer, hepatocellular carcinoma, and gastric cancer through suppression of cell proliferation, metastasis, and angiogenesis *in vitro* (7–9). Volatile oil products extracted from *Curcuma zedoaria* have been used to cure several types of hepatitis, and have also shown marked anti-inflammatory and antioxidant activity (10–12). Germacrone (**Figure 1A**) is a biologically active compound isolated from the volatile oil of *C.zedoaria* (13). Studies have shown that germacrone possesses antitumor activity; however, the mechanism underlying this effect is poorly understood. Additionally, several reports have indicated that germacrone also exhibits antidepressant, anti-inflammatory, antiulcer, antifeedant, antibacterial, antifungal, antitussive, vasodilatory, choleretic, and hepatoprotective properties (14–16).

Various tumors were shown to be sensitive to germacrone, including liver cancer, breast cancer, and glial cell carcinoma (17–19). Germacrone can induce cell cycle arrest and apoptosis by down-regulating cyclin-B1, CDK1, and Bcl-2 and up-regulating BAX, p53, and p21 (20).

Based on these observations, we hypothesized that germacrone could exert an ameliorating effect on gastric cancer. In this study, we used proteomics to identify putative molecular targets of germacrone in gastric cancer. A total of 596 protein candidates were screened, and the top hit was identified as hepatitis B X-interacting protein (HBXIP), a protein that is highly expressed in several types of human cancer (21–23). We found that HBXIP played an important role in the regulation of the cell cycle, apoptosis, and autophagy in gastric cancer cells, and that germacrone exerted its antitumor activity by acting as an antagonist of HBXIP.

## Materials and Methods

### Cell Culture and Germacrone Treatment

Human gastric adenocarcinoma SGC7901 and MGC803 cells (Shanghai Institute of Cell Biology, Chinese Academy of Sciences) were grown in DMEM supplemented with 10% fetal bovine serum (Zhejiang Tianhang Biotechnology, Hangzhou, China) in a humidified atmosphere containing 5% CO_2_ at 37°C. Cells in the exponential growth phase were used in the experiments. The experiments were divided into four groups: controls, DMSO (Beyotime Biotechnology, Shanghai, China), germacrone (CAS: 6902-91-6; Chengdu Herbpurify Co., Ltd, China), and Baf A1 (0.01 µM) or rapamycin (Rap, 0.1 µM). For Baf A1 or Rap and germacrone cotreatment, SGC7901 and MGC803 cells were first incubated with Baf A1 or Rap for 4 h, and then germacrone was added.

### MTT Assay

Plated SGC7901 and MGC803 cells were cultured in the presence or absence of germacrone (0, 50, 100, 150, 200, 250, and 300 μM) for 24, 48, and 72h. Proliferation was measured by MTT assay (Beyotime Biotechnology) to determine the effects of germacrone on cell proliferation. After germacrone treatment, MTT solution (5 mg/ml in PBS) was added (20 μl/well) and the plates incubated for another 4 h at 37°C. The formed purple formazan crystals were dissolved in 150 μl/well of DMSO. After 10 min, the absorbance at 490 nm was read using a microplate reader (Biotech Instruments, Dusseldorf, Germany).

### LDH Assay

SGC7901 and MGC803 cells were treated with germacrone for 24 h. Cytotoxic activity was determined by a standard lactate dehydrogenase (LDH) release assay. The percentage of cytotoxicity was calculated as described by the manufacturer (Beyotime Biotechnology).

### Colony Formation Assay

SGC7901 or MGC803 cells (1,000/well) were cultured for 24 h in the presence or absence of germacrone. The medium was changed and the culture continued until clear cell colonies were formed. Cells were then stained using the Wright-Giemsa Stain Kit (Nanjing Jiancheng Bioengineering Institute, China).

### Western Blot Analysis

The proteins were separated by SDS–PAGE and transferred onto polyvinylidene difluoride (PVDF) membranes (Millipore, Massachusetts, USA) by electroblotting. The membranes were incubated overnight at 4°C with antibodies against GAPDH (Cat. No. ab128915, Abcam, Massachusetts, USA), KI67 (Cat. No. ab197234, Abcam), cyclin-D1 (Cat. No. ab134175, Abcam), CDK4 (Cat. No. ab108357), CDK2 (Cat. No. ab32147, Abcam), cyclin-E1 (Cat No. ab33911, Abcam), cleaved caspase-3 (Cat. No. 11648-2-AP, Proteintech, Wuhan, China), p62 (Cat. No. ab109012, Abcam), LC3 I/II (Cat. No. D3U4C, Cell Signaling Technology, Boston, USA), ANXA 1 (Cat. No. BA3701, Boster Biological Technology, Wuhan, China), HSP70 (Cat. No. BA0928, Boster Biological Technology), and HBXIP (Cat. No. 14492-1-AP, Proteintech). The proteins were detected with an ECL chemiluminescence detection kit.

### Immunohistochemistry

Paraffin-embedded sections were dewaxed at high temperature using xylene and ethanol. Antigen retrieval was performed by boiling in sodium citrate solution, following which the samples were incubated with an anti-HBXIP antibody at 4°C overnight. After incubating the biotin-labeled goat anti-mouse/rabbit IgG and horseradish enzyme-labeled streptavidin working solution in this order, Staining was revealed with DAB (ZSGB-BIO, Beijing, China). Stained sections were observed under a microscope and imaged. Staining values were calculated according to the semi-quantitative integration method. For to the proportion of observed positive cells, the values 0,1,2,3, and 4 represented ≤5%, 5%–25%, 26%–50%, 51%–75%, and ≥75% positive cells, respectively. The assigned values were then multiplied by the staining intensity (0 = no staining, 1 = weak staining [light yellow], 2 = moderate staining [yellow-brown], or 3 = strong staining [brown]). Value between 0 and 3 were considered as low expression, while a score >3 was considered as high expression.

### Flow Cytometry Analysis

Flow cytometry (FCM) analysis was carried out on SGC7901 and MGC803 cells. For cell cycle analysis, cells were stained with propidium iodide (PI). DNA content was analyzed by FAC Scan. For the cell apoptosis analysis, cells were first stained with Annexin V–FITC and PI and detected by FCM. (The Cell Cycle and Cell Apoptosis Kits were both from BestBio, Shanghai, China).

### Hoechst 33258 Staining

Cells were seeded on sterile slides, cultured and treated, and the medium discarded. After washing 3 times with PBS, cells were fixed in 4% paraformaldehyde for 10 min. The appropriate amount of Hoechst 33258 (Beyotime Biotechnology) staining solution was added and the samples were incubated for 5 min in the dark. After washing 3 times with PBS, the plate was protected from light, and observed and imaged under a fluorescence microscope.

### Scanning Electron Microscopy

Cells were centrifuged and collected, washed 3 times with PBS, and the supernatant discarded. To fix the cell morphology, 4% glutaraldehyde was slowly added and the cells were incubated overnight at 4°C. The next day, the cells were incubated in ethanol (50, 75, 90, and 100%), air dried, and coated with gold. Cell morphology was then assessed by SEM.

### Proteomic and Bioinformatic Analyses

After treating MGC803 cells with 0.15% DMSO and 150 µM germacrone for 24 h, total protein was extracted. Protein lysates were precipitated overnight with 4 times volume of cooled acetone. The next day, the acetone was discarded and the lysates incubated with DTT (10 mM) and IAM (30 mM). Proteins were cleaved into peptides by adding 1 µg/µl trypsin (Promega, Wisconsin, USA). The collected enzymatic hydrolysates were desalted with a ZipTip pipette (Thermo Fisher Scientific, Massachusetts, USA) and concentrated in a freeze-dryer (Labconco, Kansas, USA). The precipitates were reconstituted with 0.1% formic acid.

LC–MS/MS: A nano-ESI HPLC system (Dionex, Thermo Fisher Scientific) equipped with a C18 column (5 μm, 100 mm × 0.1 mm, 300 A) was used for separation, and the injection volume was 4 μg/8 μl. Mobile phase A was 5% acetonitrile (ACN) (buffer A, containing 0.1% formic acid) and mobile phase B was 95% ACN (buffer B, containing 0.1% formic acid). The gradient elution program was as follows: 0–5 min, 5% buffer B; 5–45 min, 5%–35% buffer B; 45–50 min, 35%–90% buffer B; 50–55 min, 90% buffer B; 55–56 min, 90%–5% buffer B; 56–65 min, 5% buffer B. Q-Exactive (ThermoFisher Scientific): Intact peptides were detected in the Orbitrap at a resolution of 70,000 FWHM. Peptides were selected for MS/MS using high-energy collision dissociation (HCD) operating mode with a normalized collision energy setting of 27%; ion fragments were detected in the Orbitrap at a resolution of 17,500 FWHM. A data-dependent procedure that alternated between one MS scan followed by 15 MS/MS scans was applied for the 15 most abundant precursor ions above a threshold ion count of 20,000 in the MS survey scan with an ensuing Dynamic Exclusion duration of 15 s. The electrospray voltage applied was 1.6 kV. Automatic gain control (AGC) was used to optimize the spectra generated by the Orbitrap. The AGC target for full MS was 3e6 and 1e5 for MS2. For MS scans, the *m/z* scan range was 350 to 2,000 Da. The RAW data generated by Q-Exactive was outputted *via* the Xcalibur workstation (Thermo Fisher Scientific).

Data was imported into Proteome Discoverer (V2.2, ThermoFisher Scientific). After searching the Human Proteome Database (2015.3.4) downloaded from the Uniprot website (ftp://ftp.uniprot.org/), the relevant information was obtained for the detected protein. After excluding redundant proteins and filtering to an FDR <1%, we matched 4014 proteins. Filter by: unique peptides >2, abundance ratio>1.2, P<0.05, we screened suitable proteins. Filter by: unique peptides >2, abundance ratio >1.2, P<0.05, we screened suitable proteins.

### Reverse Transcription–Polymerase Chain Reaction

RNA was extracted from MGC803 and SGC7901 cells using the TRIzol reagent (Life Technologies, Burlington, Ontario, Canada) and purified by sequential washing with chloroform, isopropanol, and 75% ethanol. The cDNA was synthesized by using Superscript First-Strand Synthesis System kits (Invitrogen Life Technologies Carlsbad, CA, USA). HBXIP forward 5’-CGAGGTTTGCGGTGAAGG-3’and reverse 5’-CCACGGCAGCCCAGATTA-3’; GAPDH forward 5’-GAAGGTGAAGGTCGGAGTC-3’, and reverse 5’-GAAGATGGTGATGGGATTTC-3’. The sequences were amplified according to the instruction (Vazyme, Nanjing, China).

### Small Interfering RNA Transfection to HBXIP

si-RNA directed against HBXIP (si-HBXIP-1 and si-HBXIP-2) were supplied by Life Technologies (GenePharma, China). MGC803 and SGC7901 cells were transfected with 20 nM oligonucleotide using Lipofectamine™ RNAiMAX (Life Technologies). HBXIP siRNA sense-1: CGGAAGCGCAGUGAUGUUUdTdT; HBXIP siRNA sense-2: GCAGCUAAGGCAGCUAAGCUAACCUCUGTT.

### Immunofluorescence

Cells were seeded on sterile slides, cultured and treated. Cells were fixed with 2% paraformaldehyde for 10 min and blocked by 5% BSA for 30 min, then incubated with anti-LC3 antibody at 4°C overnight. The appropriate anti-FITC (Beyotime Biotechnology) staining solution was added and covered the cells 1 h in the dark. Observed and imaged under the fluorescence microscope.

### Statistical Analyses

All experiments in this study were repeated more than 3 times and the data were presented as means ± standard deviation. Statistical analysis was performed using SPSS 17.0. Comparisons between multiple groups were performed by one-way analysis of variance. Comparisons between two groups were performed using Dunnett’s *t*-test. Results were considered significant if *P*<0.05.

## Results

### Germacrone Inhibited the Proliferation of Gastric Cancer Cells in a Dose-Dependent Manner

Following germacrone treatment, SGC7901 and MGC803 cell viability was significantly inhibited in a dose-dependent manner (**Figures 1B–D**), whereas cytotoxicity was increased, also in a concentration-dependent manner (**Figure 1E**). The colony-formation assay demonstrated that germacrone could inhibit the proliferative ability of gastric cancer cells (**Figures 1F, G**). Immunofluorescence and western blotting were used to detect the expression of KI67. The results showed that KI67 expression was decreased with germacrone treatment (**Figures 1H, I**). These results indicated that germacrone can inhibit the proliferation of gastric cancer cells.

### Germacrone Induced G0/G1-Phase Cell Cycle Arrest in Gastric Cancer Cells

**Figure 2:**
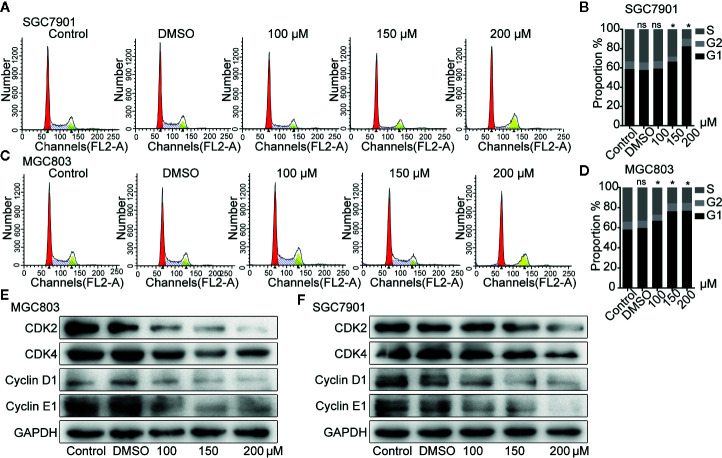
Germacrone induced G0/G1-phase cell cycle arrest in gastric cancer cells. **(A–D)** Propidium iodide (PI) staining was used for flow cytometric (FCM) analysis of the effect of germacrone on the cell cycle. **(E, F)** Western blot was used to detect changes in the expression levels of CDK2, CDK4, cyclin-D1, and cyclin-E1. Data are the means ± SD of three experiments. *P < 0.05; ns, not significant.

Flow cytometry analysis showed that SGC7901(**Figures 2A, B**) and MGC803 (**Figures 2C, D**) cells in the G0/G1 phase gradually accumulated after treatment with 100, 150, and 200 µM germacrone for 24 h. Consistent with the flow cytometry analysis, the western blot results showed that G0/G1-related proteins such as cyclin-D1, cyclin-E1, CDK2, and CDK4 were gradually downregulated in the germacrone groups (**Figures 2E, F**).

### Germacrone Induced Apoptosis in Gastric Cancer Cells

**Figure 3:**
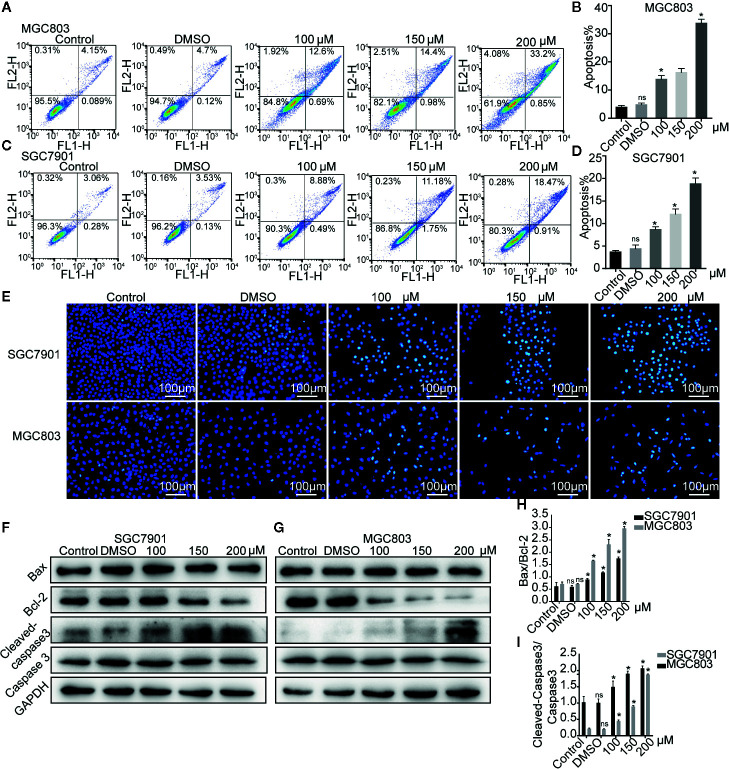
Germacrone induced apoptosis in gastric cancer cells. **(A–D)** Annexin IV-fluorescein isothiocyanate (FITC)/propidium iodide (PI) staining was used to assess the effect of germacrone on apoptosis by flow cytometry (FCM). **(E)** Hoechst 33258 staining was used to detect apoptosis. **(F, G)** Changes in the expression levels of BAX, Bcl-2, caspase-3, and cleaved caspase-3 were detected by western blot. **(H, I)** Changes in the BAX/Bcl-2 and cleaved caspase-3/caspase-3 ratios were analyzed after germacrone treatment. Data are the means ± SD of three experiments. *P < 0.05; ns, not significant.

The effect of germacrone on apoptosis was also investigated. As shown in **Figures 3A–D**, the apoptotic rate with germacrone treatment at 100, 150, and 200 µM was significantly higher than that in the control group. Hoechst 33258 staining further showed an increase in the number of apoptotic cells with germacrone treatment group (**Figure 3E**). The ratio of cleavedcaspase-3/caspase-3 (**Figures 3F–H**) increased significantly with increasing germacrone concentrations. Moreover, Bcl-2 expression was gradually decreased, and the BAX/Bcl-2 ratio was also significantly enhanced (**Figure 3I**), further indicating that germacrone treatment promoted a gradual increase in cell apoptosis.

### Germacrone Promoted the Formation of Autophagosomes in Gastric Cancer Cells

**Figure 4:**
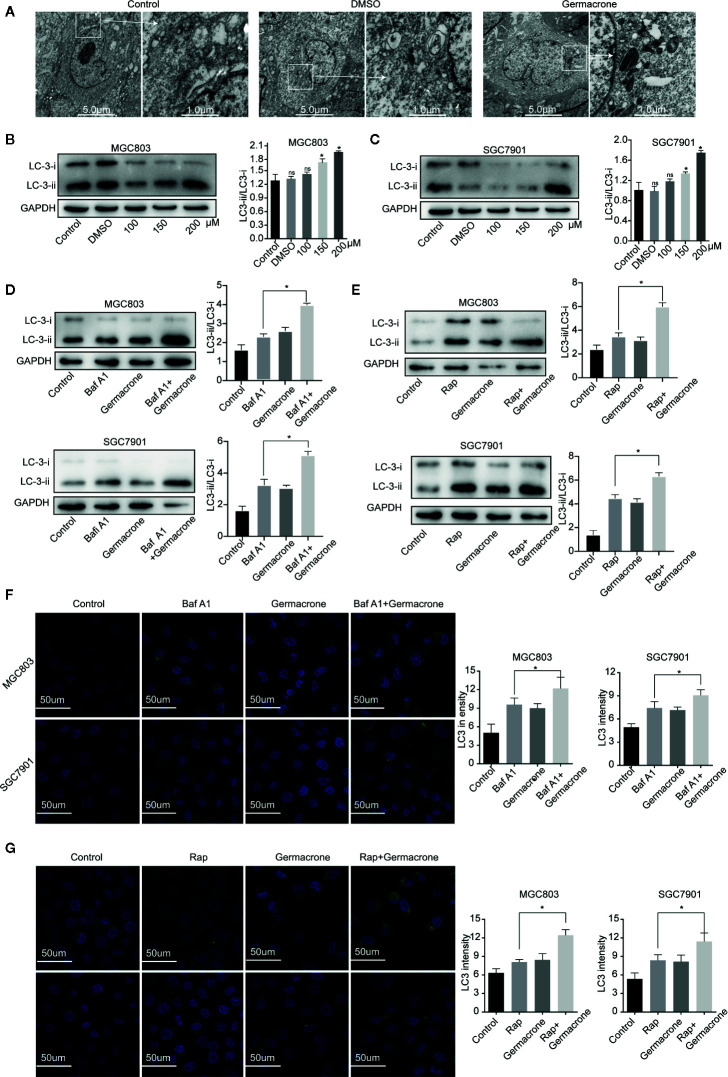
Germacrone promoted the formation of autophagosomes in gastric cancer cells. **(A)** Scanning electron microscopy was used to visualize autophagosome accumulation. **(B, C)** Changes in the protein expression levels of LC3I, LC3II were detected by western blot. **(D)** Changes in the protein expression levels of LC3I, LC3II in SGC7901 and MGC803 cells after 24 h of treatment with Baf A1. **(E)** Changes in the protein expression levels of LC3I, LC3II in SGC7901, and MGC803 cells after 24 h of treatment with Rap. **(F)** Immunofluorescence was used to detect the protein expression levels of LC3 after exposure to Baf A1 and germacrone (150 µM) for 24 h. **(G)** Immunofluorescence was used to detect the protein expression levels of LC3 after exposure to Rap and germacrone (150 µM) for 24 h. Data are the means ± SD of three experiments. *P < 0.05; ns, not significant.

Autophagy provides nutrients to cells, but excessive autophagy also accelerates autophagic cell death. Electron microscopy analysis revealed that the autophagosomes was increased after the germacrone treatment compared with control in MGC803 (**Figure 4A**). The expression of LC3II and LC3II/LC3I also increased rapidly in gastric cancer cells treated with germacrone (**Figures 4B, C**). Baf A1 is an autophagy inhibitor, which affects the binding of autophagosomes to lysosomes, and the increasing of LC3 (24, 25). In our study, we used Baf A1 to investigate the regulatory target of germacrone on autophagy. The combined effect of Baf A1 and germacrone on LC3II/LC3I was significant increased compared with that in germacrone in MGC803 and SGC7901 (**Figure 4D**). The combination of Rap, as an autophagy inducer, and germacrone could increase LC3II/LC3I compared with Rap or gemmazone treating alone (**Figure 4E**). The results of immunofluorescence were consistent with these results (**Figures 4F, G**). The results suggested that germacrone promoted the formation of autophagosomes.

### Label-Free Proteomic and Bioinformatic Analysis Identified Proteins Associated With the Cell Cycle, Apoptosis, and Autophagy in Gastric Cancer Cells

**Figure 5:**
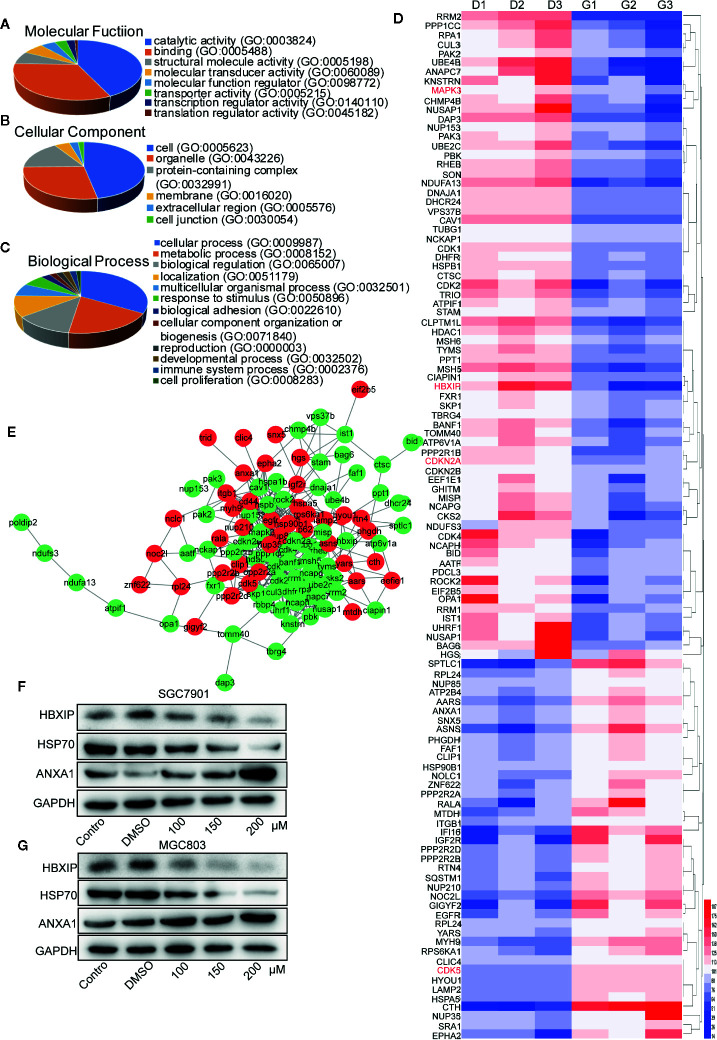
Label-free proteomic and bioinformatic analysis of proteins associated with the cell cycle, apoptosis, and autophagy. **(A–C)** The DAVID database was used for analysis of molecular function, cellular component, and biological process. **(D)** A heat map was constructed based on the abundance of 111 proteins. D1, D2, and D3 represent the DMSO 1, DMSO 2, and DMSO 3 groups; G1, G2, and G3 represent the germacrone 1, germacrone 2, and germacrone 3 groups. **(E)** A protein interaction network was constructed in Cytoscape based on the information provided by the STRING database. Red indicates increased expression of the protein in the germacrone group; green indicates reduced expression of the protein in the germacrone group. **(F, G)**. The expression of HBXIP, HSP70, and ANXA1 was detected to verify the accuracy of the proteomics results.

Five hundred ninety-six suitable proteins among the 4,014 proteins have been identified. A total of 350 proteins were down-regulated and 246 up-regulated in the germacrone-treated group compared with the DMSO group. Analysis using the Database for Annotation, Visualization, and Integrated Discovery (DAVID; https://david.ncifcrf.gov/) identified 111 proteins related to the cell cycle, apoptosis, and autophagy. These proteins were analyzed for molecular function, cellular component, and biological function (**Figures 5A–C**). The 111 proteins comprised mainly hydrolases, nucleic acid binding proteins, and transferases that are involved in the p53 and Ras signaling pathways. Heat map analysis showed that the expression of these proteins was significantly different in the groups of germacrone and DMSO (**Figure 5D**). The 111 proteins were introduced into the STRING database (https://string-db.org/cgi/input.pl) and Cytoscape to analyze protein interactions and construct an interaction network (**Figure 5E**). Combined with the changes in CDK2, CDK4, and p62 expression, the up-regulation of ANXA1 and downregulation of HSP70expression detected by western blot were consistent with the proteomic results, thereby confirming the accuracy of the results (**Figures 5F, G** **)**.

We identified four proteins involved in the cell cycle, apoptosis, and autophagy: cyclin-dependent-like kinase 5 (CDK5), cyclin-dependent kinase inhibitor 2A (CDKN2A), mitogen-activated protein kinase 3 (MAPK3), and late endosomal/lysosomal adaptor and MAPK and MTOR activator 5 (LAMTOR5, also named HBXIP) (**Table 1**). Based on the magnitude of the difference, we selected HBXIP for further analysis. In our validation experiments, the expression of HBXIP was significantly downregulated by germacrone treatment. At the same time, germacrone also reduced HBXIP at the transcription level (**Figures S1A, B**).

**Table 1:** Details of four proteins related to the cell cycle, apoptosis, and autophagy.

Name	Accession(Uniprot)	Description	Coverage[%]	# AAs	MW [kDa]	Calc. pI	Score Sequest HT	Expression Change with germacrone treatment
LAMTOR5	O43504	Regulator complex protein LAMTOR5 OS=*Homo sapiens* GN=LAMTOR5 PE=1 SV=1	37	91	9.6	4.87	8.85	Down
MAPK3	P27361	Mitogen-activated protein kinase 3 OS=*Homo sapiens* GN=MAPK3 PE=1 SV=4	4	379	43.1	6.74	3.3	Down
CDK5	Q00535	Cyclin-dependent-like kinase 5 OS=*Homo sapiens* GN=CDK5 PE=1 SV=3	8	292	33.3	7.66	8.7	Up
CDKN2A	P42771	Cyclin-dependent kinase inhibitor 2A OS=*Homo sapiens* GN=CDKN2A PE=1 SV=2	59	156	16.5	5.81	71.16	Down

OS, organism name; GN, gene name; PE, protein existence; SV, sequence version; MW, molecular weight; #AAs, number of amino acids; Calc. pI, calculated isoelectric point.

### HBXIP Highly Expressed in Gastric Cancer and Involved in the Germacrone Regulation

**Figure 6:**
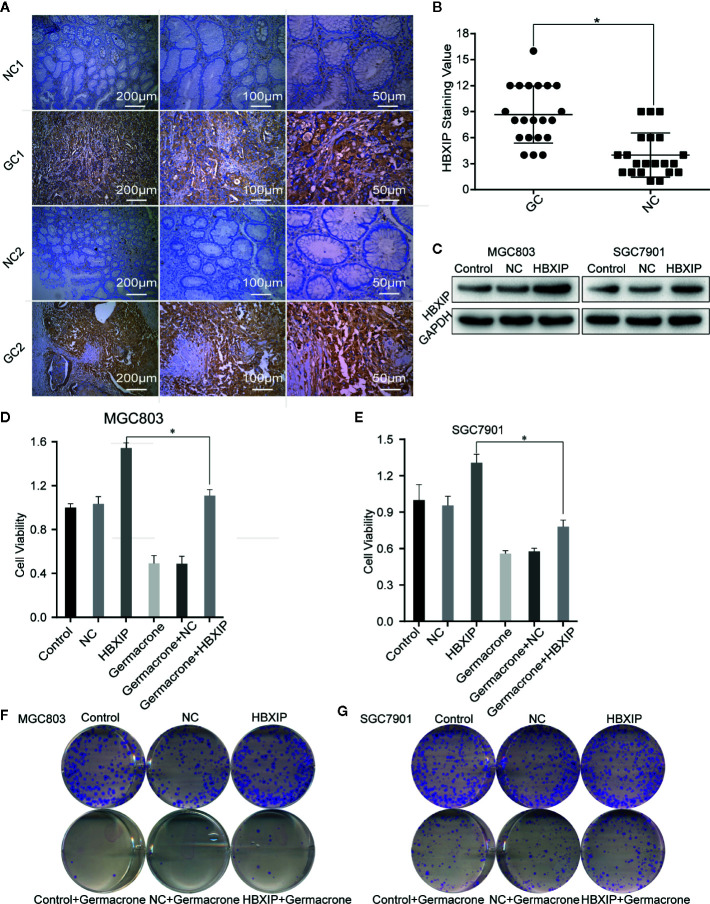
HBXIP highly expressed in gastric cancer and involved in the germacrone regulation. **(A, B)** Immunohistochemistry was used to detect the expression of HBXIP in tissue from gastric cancer patients and matched adjacent tissues. **(C)** A HBXIP overexpression model was constructed in gastric cancer cells. **(D, E)** An MTT assay was used to evaluate the effects of germacrone and overexpression of HBXIP on cell viability. **(F, G)** A colony formation assay was used to assess the effects of germacrone and overexpression of HBXIP on cell viability. *P < 0.05.

We collected cancerous and matched paracancerous tissues from 21 gastric cancer patients following surgical resection, and found that HBXIP was expressed in gastric cancer tissue from all the patients. What’s more, we found that HBXIP expression was significantly up-regulated in gastric cancer tissues (**Figures 6A, B**). Overexpression of HBXIP was induced successfully in MGC803 and SGC7901 cells (**Figure 6C**). Overexpression of HBXIP enhanced proliferation in MGC803 and SGC7901 cells. Treated with germacrone in overexpressed-HBXIP cells, the surviving rate and colony formation of SGC7901 and MGC803 decreased significantly (**Figures 6D–G**). Treated with germacrone in si-HBXIP cells, the results showed that si-HBXIP inhibited the proliferation of gastric cancer cells and the inhibitory effect was not significant combined with germacrone (100 μM). Compared with the si-HBXIP, the combination of germacrone (150 μM) with si-HBXIP had a stronger inhibitory effect on the proliferation of gastric cancer cells. (**Figures S2A–B**).

### Overexpression of HBXIP Regulated Autophagy and Reversed the Germacrone-Induced Cell Cycle Arrest and Apoptosis

**Figure 7:**
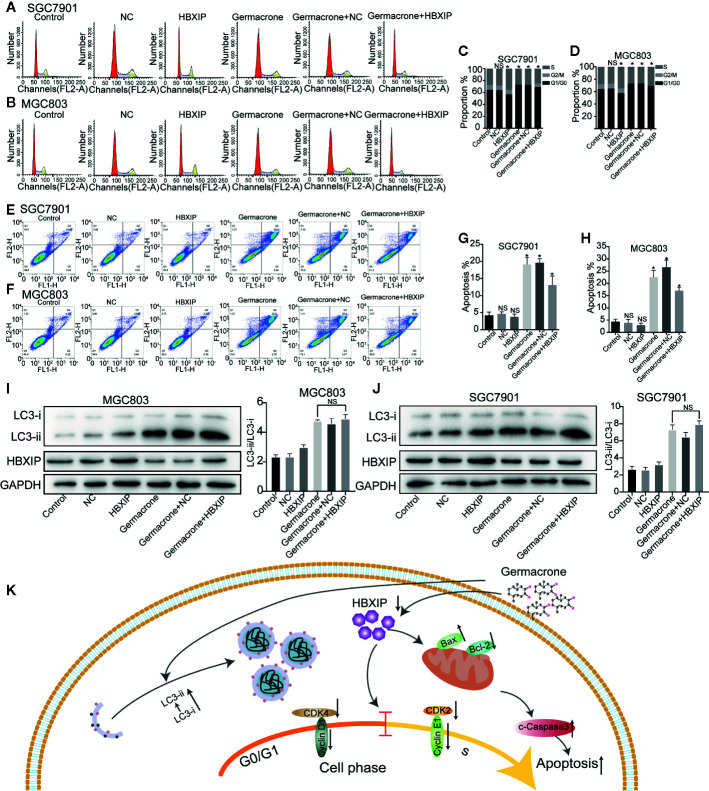
Overexpression of HBXIP regulated autophagy and reversed the germacrone-induced cell cycle arrest and apoptosis. **(A–D)** Propidium iodide (PI) staining was used for flow cytometric (FCM) analysis of the effect of germacrone and overexpression of HBXIP on the cell cycle. **(E–H)** Annexin IV-fluorescein isothiocyanate (FITC)/PI staining was used to detect the effect of germacrone and overexpression of HBXIP on apoptosis by FCM. **(I, J)** The expression of HBXIP, p-62, LC3I, and LC3II was detected by western blot. **(K)** A schematic of the effect of germacrone on gastric cancer cells. Germacrone inhibits gastric cancer cell proliferation involving HBXIP-mediated regulation of the cell cycle, apoptosis, and autophagy. Data are the means ± SD of three experiments. *P < 0.05; ns, not significant.

We used FCM to detect changes in the cell cycle and cell apoptosis. The number of MGC803 and SGC7901 cells in the S-phase was significantly increased when HBXIP was overexpressed, overcoming the G0/G1 phase arrest induced by germacrone (**Figures 7A–D**). Germacrone treatment induced apoptosis in SGC7901 and MGC803 cells; however, this effect was reduced with overexpression of HBXIP (**Figures 7E–H**). At the level of autophagy, overexpression of HBXIP weakly enhanced the expression of LC3II compared with the control group. Combining with germacrone did not reverse these effects when compared with germacrone treatment alone (**Figures 7I, J**).

## Discussion

Germacrone [(3E,7E)-3,7-dimehyl-10-propan-2-ylidenecyclodeca-3,7-dien-1-one] is a well-characterized anticancer terpenoid isolated from *Curcuma zedoaria*. Zhong et al. (26), reported that germacrone inhibited the proliferation of breast cancer cells *in vitro* by inducing cell cycle arrest and apoptosis. This antiproliferative effect of germacrone was associated with apoptosis, G2/M-phase cell cycle arrest, and a decrease in the levels of cyclin-B1 and its activation partner CDK1, which resulted in the upregulation of p21 and p53 levels and enhancement of ROS production (19). Germacrone was also shown to down-regulate JAK2/STAT3activation, modulate the expression of p53 and Bcl-2 family members, and induce apoptosis in HepG2 cells (22). Additionally, germacrone has been reported to be effective as a treatment for liver cancer. In this study, we found that germacrone can inhibit the proliferative ability of SGC7901 and MGC803 cells. When treated with germacrone, these gastric cancer cells appeared G0/G1-phase cell cycle arrest, a significantly higher degree of apoptosis, and the formation of autophagosomes increased.

Hepatitis B X-interacting protein is named for its ability to specifically bind to the carboxyl terminus of the HBx protein (27). Although relatively little is known about HBXIP, existing studies have shown that it is a multifunctional, regulatory protein involved in HBV replication, cell proliferation, apoptosis, centrosome replication, and regulation of cell division. Recent studies have reported that HBXIP is an oncogenic protein that is upregulated in a variety of cancers. HBXIP was found to be highly expressed in MCF-7 and SK-BR3 breast cancer cells. In contrast, no HBXIP expression was detected in epithelial MCF-10A breast cancer cells (28). In bladder urothelial carcinoma, inhibition of HBXIP reduced the proliferative, migratory, and invasive capacity of T24 and PC3 cells (29). Importantly, these authors observed that inhibition of HBXIP reduced tumorigenesis *in vivo*. In clinical studies, HBXIP was elevated in normal gastric mucosa, as well as in gastric cancer tissue and adjacent, noncancerous tissue (30). Other studies have also confirmed that HBXIP is highly expressed in both liver cancer and ovarian cancer (31, 32). These observations indicate that HBXIP plays an important role in the occurrence, invasion, and metastasis of malignant tumors. Here, we collected gastric cancer and paracancerous tissues from 21 patients undergoing gastric cancer resection, and found that HBXIP expression was also elevated in gastric cancer tissue, consistent with previous studies. On the contrary, knockdown-HBXIP inhibited gastric cancer cell proliferation. Combining si-HBXIP and germacrone synergistically inhibited the expression of HBXIP, which may be the cause for the more significant inhibition in proliferation. Bioinformatics analysis also indicated that HBXIP affected cell cycle, apoptosis and autophagy.

HBXIP can act as a mediator of DNA damage response signals, activating G2/M checkpoints to maintain genomic integrity and prevent cell death (33). Wang et al. (34), found that HBXIP can upregulate the expression of cyclin-D1 and cyclin-E in normal L-02liver cells, H7402 liver cancer cells, and MCF-7 breast cancer cells, and inhibit the expression of P21 and P27, which induce the G1/S-phase cell cycle transition. In HepG2 hepatocellular carcinoma cells, HBXIP activates the PI3/Akt signaling pathway, which upregulates the expression of cyclin-D1 and phosphorylated protein kinase B, and downregulates that of p53 and p21 (35). A decrease or absence of HBXIP protein expression results in an increase in the number of cells containing single-stage spindles, and most affected cells cannot divide. Conversely, high expression of the HBXIP protein results in an increase in the number of cells containing tertiary or multi-stage spindles (36). Marusawa et al. (37), found that, in HepG2 cells, HBXIP forms a complex by binding to HBX and survivin, thereby competitively inhibiting the activation of the caspase-9 precursor protein by Apaf1 and blocking mitochondria-mediated cell apoptosis. In a different study (38), the authors identified the region responsible for hSuv3p binding to HBXIP, and reported that the HBXIP binding domain is important for mitochondrial import and stability of the Suv3 protein *in vivo*. Moreover, the study also showed that the hSuv3p-HBXIP interaction may be related to survivin-dependent antiapoptotic pathways. HBXIP is a member of the Regulator complex, a pentamer consisting of p18 (LAMTOR1), p14 (LAMTOR2), MP1 (LAMTOR3), C7orf59 (LAMTOR4), and HBXIP (LAMTOR5). Among these, HBXIP-C7orf59 is responsible for the initial nucleation of the complex and stabilization of the p18 conformation, allowing the subsequent binding of MP1-p14 (39). The Regulator complex plays a key role in the activation of the mammalian target of rapamycin complex 1 (mTORC1) on the lysosomal surface, and can also regulate the branching of the MAPK pathway by recruiting MEK1 to MP1 (40). These observations indicate that loss of HBXIP may affect the formation and function of the Regulator complex. The role of HBXIP in autophagy is mainly focused on molecular chaperone-mediated autophagy (CMA) (41, 42), which has been less studied in macro autophagy. Our results showed that LC3II/LC3I increased after treatment with germacrone. Compared with germacrone, the combined effect of Baf A1 or Rap and germacrone on LC3II/LC3I was significant increased, which suggest that germacrone increased the formation of autophagosomes. After overexpressing HBXIP, LC3-II increased. The overexpression of HBXIP did not significantly change the effect of germacrone on the increase of LC3II/LC3I. It may suggest that HBXIP does not play a key role in the process of the formation of autophagosomes increased by germacrone. Chen et al, found that germacrone plays an anti-oxidative effect (43), and a high degree of oxidative stress damages the promoter of P62 and inhibits P62 expression (44). We found the expression of P62 increased after germacrone treatment (**Figures S3A, B**). We speculate that the effect of germacrone was related to the oxidative stress process.

## Conclusion

Treating gastric cancer cells with germacrone resulted in cell cycle arrest, apoptosis, and formation of autophagosomes. The effect of germacrone is related to its inhibitory effect on HBXIP. The effect on cell cycle and apoptosis of germacrone was effectively alleviated after overexpressing of HBXIP. Based on this result, we concluded that germacrone inhibits gastric cancer cell proliferation by inhibiting HBXIP-mediated regulation of the cell cycle and apoptosis. We mainly draw the following conclusions: Germacrone can regulate apoptosis, cell cycle and autophagy in gastric cancer cell; HBXIP is a potential target for Germacrone in treating gastric cancer. However, the mechanism of HBXIP regulating these processes remains unknown, and it is the direction of our subsequent research (**Figure 7K**).

## Data Availability Statement

The datasets presented in this study can be found in online repositories. The names of the repository/repositories and accession number(s) can be found below: ProteomeXchange Consortium (http://proteomecentral.proteomexchange.org) *via* the iProX partner repository: PXD021980.

## Ethics Statement

The studies involving human participants were reviewed and approved by the Committee on Medical Ethics of the First Affiliated Hospital of Anhui Medical University. Written informed consent to participate in this study was provided by the participants’ legal guardian/next of kin.

## Author Contributions

XF and TT designed the project. BG performed the experiments. YZ participated in the bioinformatic analysis. QX and TL optimized the manuscript. All authors contributed to the article and approved the submitted version.

## Funding

This work was supported by the National Natural Science Foundation of China (No 81903622), the National Natural Science Foundation of China (No 81273245) and the Natural Science Foundation of Anhui Province, China (1908085QH378).

## Conflict of Interest

The authors declare that the research was conducted in the absence of any commercial or financial relationships that could be construed as a potential conflict of interest.

## Supplementary MateriaL

Supplementary Figure 1 Germacrone regulated HBXIP transcription level. **(A)** After treatment of MGC803 cells with 100, 150, 200 μM germacrone, RT-PCR detected changes in HBXIP RNA levels.

Supplementary Figure 2 Knocked down HBXIP decreased cell proliferation. **(A)** Western blot experiments verified the knockdown effect of si-RNA. **(B)** MTT assay was used to evaluate the effects of germacrone and knockdown of HBXIP on cell viability.

Supplementary Figure 3 Germacrone increased the expression of P62. **(A, B)** Western blot experiments detected the expression of P62 after the treatment of germacrone in SGC7901 and MGC803.
