# Supplementary figures and images for: Germacrone Regulates HBXIP-Mediated Cell Cycle, Apoptosis and Promotes the Formation of Autophagosomes to Inhibit the Proliferation of Gastric Cancer Cells
Source: Front Oncol. 2020 Nov 10;10:537322. doi: 10.3389/fonc.2020.537322 (PMC7683780; doi:10.3389/fonc.2020.537322)

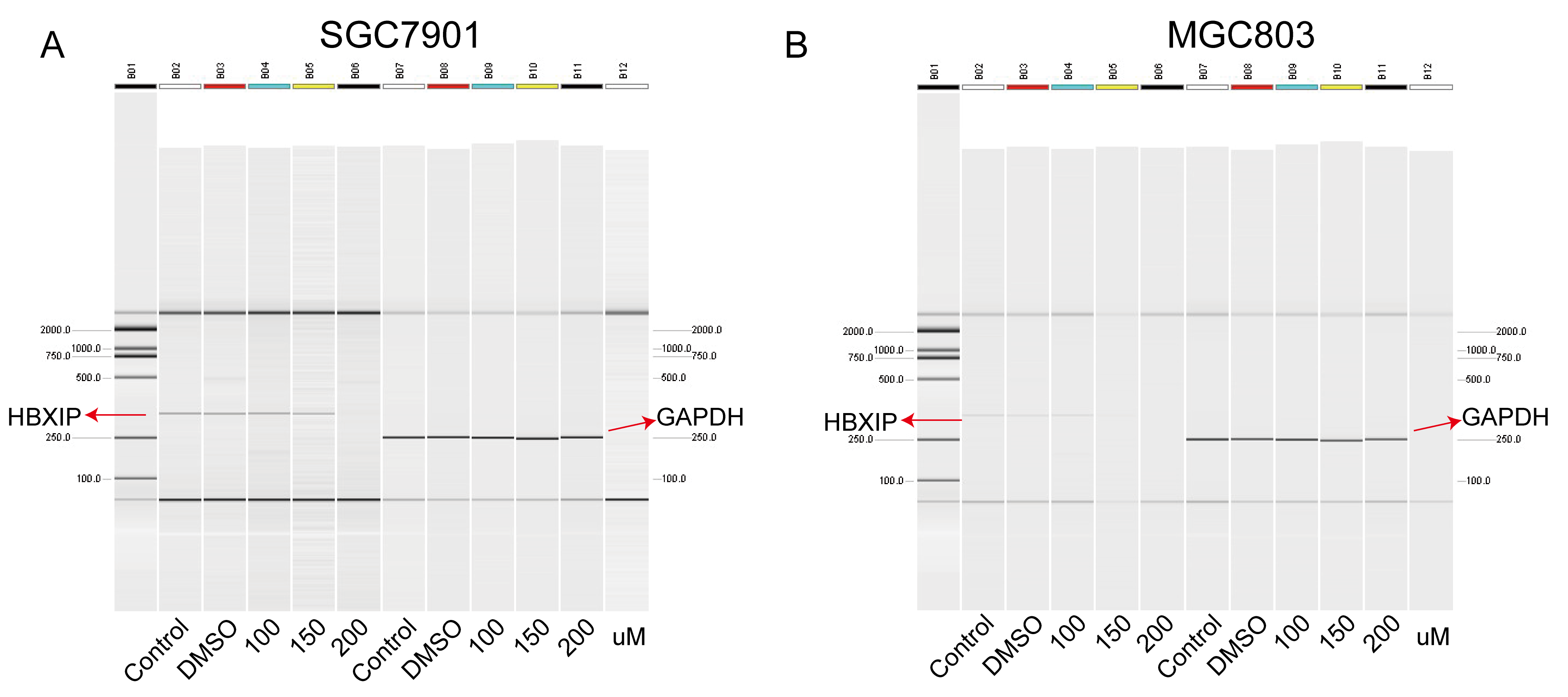

Supplement: Supplementary Figure 1 — Germacrone regulated HBXIP transcription level. (A) After treatment of MGC803 cells with 100, 150, 200 μM germacrone, RT-PCR detected changes in HBXIP RNA levels. [file Image_1.tif]

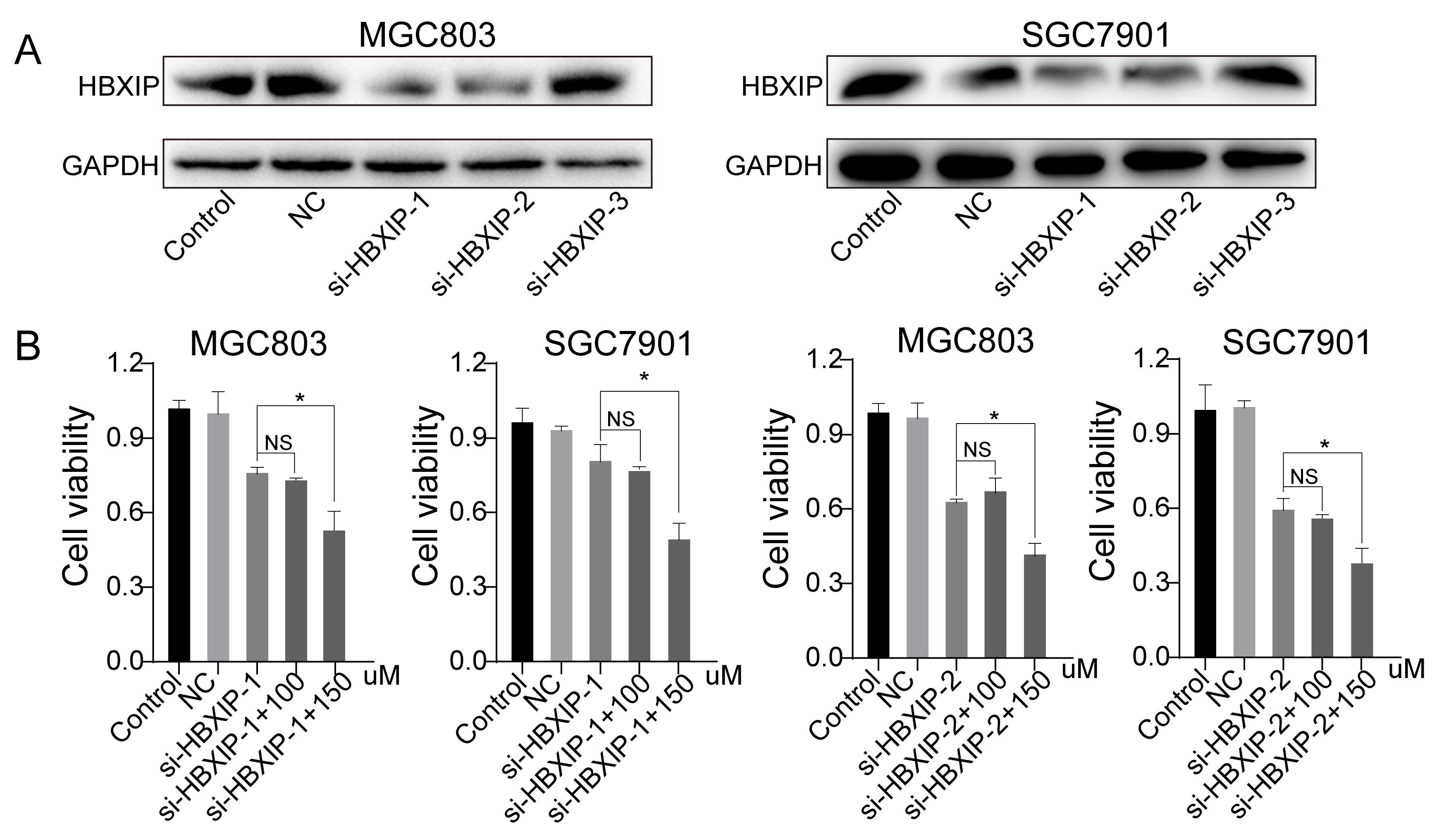

Supplement: Supplementary Figure 2 — Knocked down HBXIP decreased cell proliferation. (A) Western blot experiments verified the knockdown effect of si-RNA. (B) MTT assay was used to evaluate the effects of germacrone and knockdown of HBXIP on cell viability. [file Image_2.tif]

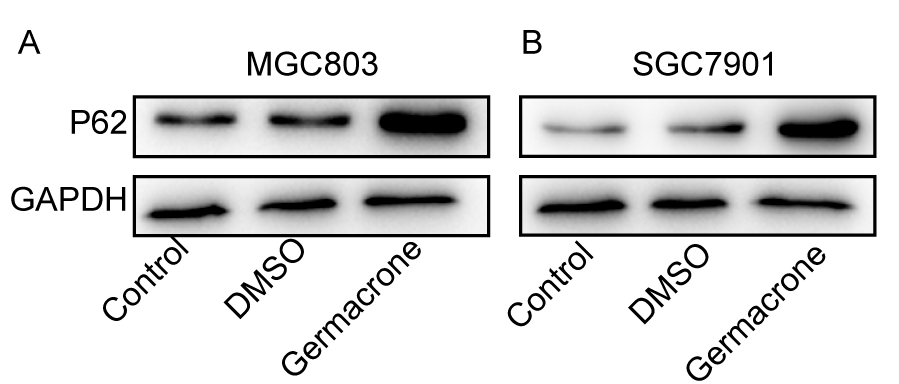

Supplement: Supplementary Figure 3 — Germacrone increased the expression of P62. (A, B) Western blot experiments detected the expression of P62 after the treatment of germacrone in SGC7901 and MGC803. [file Image_3.tif]
